# Progression of the Pathway for Public Health Care during the COVID-19 Outbreak at District Health Office

**DOI:** 10.3390/ijerph181910533

**Published:** 2021-10-07

**Authors:** Noraziani Khamis, Intan Syafinaz Saimy, Nor Hayati Ibrahim, Nur Khairah Badaruddin, Nor Zam Azihan Mohd Hassan, Faridah Kusnin, Sukhvinder Singh Sandhu, Masitah Mohamed

**Affiliations:** 1 Institute for Health Management, Ministry of Health Malaysia, Shah Alam 40170, Malaysia; intan.syafinaz@moh.gov.my (I.S.S.); drnorhayatii@moh.gov.my (N.H.I.); nurkhairah.b@moh.gov.my (N.K.B.); 2 Institute for Health Systems Research, Ministry of Health Malaysia, Shah Alam 40170, Malaysia; norzam@moh.gov.my; 3 Klang District Health Office, Selangor Health Department, Ministry of Health Malaysia, Klang 41200, Malaysia; frdhkusnin@gmail.com (F.K.); sukhyaki1@yahoo.com (S.S.S.); dr.masitah@moh.gov.my (M.M.)

**Keywords:** district health office, public health activities, COVID-19 control and prevention, COVID-19 outbreak

## Abstract

Public health activities under district health offices (DHOs) play a major role in Malaysia’s fight against COVID-19. This article aims to describe and illustrate the public health activity pathway in combating the COVID-19 pandemic, and a team of public health workers who are familiar with DHO work settings was created in April 2020 for that purpose. Review of documents and the Ministry of Health’s updates was carried out, followed by a series of discussions with stakeholders. Based on the steps in the outbreak investigation tasks, the flow of activities from January to May 2020 was listed in line with the phases of the country’s National Movement Control Order 2020. Results show that the activities can be classified into three different sections—namely, the main action areas, category of cases, and level of care. The main process flow of activities comprised the case management and support activities. Case management flow was split into tasks for patients under investigation and persons under surveillance, while the support services existed throughout the phases. The pathways illustrate that the progression of the pandemic translated directly to changes in the pattern of activities, with additional subgroups of activities in accordance with all imposed guidelines.

## 1. Introduction

The COVID-19 outbreak has rapidly extended globally, and has become a major public health burden [1,2,3]. Globally, COVID-19 has caused over 20.5 million years of life lost (YLL) [4]. The burden of the ongoing pandemic, extensively quantified by the adoption of the disability-adjusted life years (DALYs) composite indicator [5,6,7,8], has had burdensome consequences, in terms of both mortality and morbidity, on societies worldwide, with hulking social and economic repercussions. The World Health Organization (WHO) defines an outbreak as “the occurrence of disease cases in excess of normal expectancy” [9]. Outbreak management consists of all activities involved in investigating and responding to outbreaks, with the aim to minimize their impact on public health. This framework has several major elements, including outbreak preparedness, surveillance, investigation, and response [10,11].

Outbreak preparedness is the first essential step, and this includes outbreak protocols, designation of coordinators and teams, and the assembly of the necessary materials for outbreak investigation and response, as well as identifying training needs that should be addressed well before the occurrence of an outbreak. A good surveillance system would allow early detection of outbreaks, leading to activities related to outbreak investigation and response [12,13,14].

Outbreaks are usually events that are considered exceptional, as they often require extra human and financial resources, while also relying on other sectors or agencies as additional partners in their management [15]. In this instance, containing a pandemic as huge as COVID-19 requires full participation at various levels from the Malaysian Ministry of Health (MOH), as well as other ministries and agencies [16,17]. Comprehensive public health measures undoubtedly play a huge role in controlling this pandemic [18,19,20].

Public health strategies have been adopted in many countries to varying degrees [21,22,23]. Among the Malaysian government’s strategies for enhancing the breaking of the chain of infection and containing the spread of the disease was a nationwide “Movement Control Order (MCO) 2020”, which was also often referred to as partial lockdown, between 18 March and 12 May 2020 [24,25,26]. This consisted of providing public education, closing schools and non-essential services, limiting public gatherings, issuing travel restrictions, screening travelers, and tightening interstate and interdistrict border control, among other measures.

Undoubtedly, Malaysia has done well in managing COVID-19, and the country’s response had proven effective as of July 2020 [24,25,27]. This is despite the many public health management difficulties due to the novelty of the disease, with limited understanding of the viral pathogenesis, risk factors, clinical presentation and outcomes, prognostic factors for severe illness, period of infectivity, transmission, effective preventive measures, vaccines, and containment interventions.

These uncertainties pose a challenge to health authorities worldwide, but in Malaysia—and especially in the district health offices (DHOs), also generally known as *Pejabat Kesihatan Daerah*, which are the center of activity for public health responses at the district level—they persevere. A wide distribution network of DHOs and public hospitals of the government of Malaysia provide a comprehensive range of services covering disease prevention and curative care. Apart from DHO services pertaining to maternal and child health care, vaccination, home visits, and postnatal care, other existing organized public health tasks include regulating and monitoring health programs to prevent the occurrence or spread of infectious diseases such as measles, dengue, leptospirosis, and food poisoning, as well as environmental and occupational health care, and many more [28]. As a rule, in Malaysia, notification of an infectious disease is in accordance with the Prevention and Control of Infectious Disease Act 1988, which makes it compulsory for any health provider to notify the nearest district health officer of any infectious disease as soon as it is made known to the provider [29].

DHOs are distributed in more than 130 districts in Malaysia, with health clinics operating under their purview [30]. A DHO functions as the basic operational level in Malaysia’s health care system [28]. DHOs manage resources and data for the public health services delivered to the community within a district [14], and they interact with various stakeholders in planning and executing activities.

To better understand the pattern of activities in a DHO, it can be illustrated using a care pathway. Care pathways are one of several quality improvement tools to address issues related to patient safety and standards of care [31]; they include a set of interventions for a specific condition [32,33]. Care pathways may potentially improve service delivery by connecting all activities with a defined service objective. In managing a care unit or a DHO, the applied pathway will assist in shortening the service by starting the activities sooner or increasing parallel activities [33].

Based on this concept, the present report aims to describe and illustrate the public health measures pathway in combating the COVID-19 pandemic, specifically by describing the different kinds of activities performed at a DHO, along with the changes in the flow of activities over a period of time. A care pathway is proposed to reflect most of the public health services, in contrast to a curative care pathway, which is mostly concentrated in hospital settings [34,35,36]. However, with regard to the methods and systems of DHOs, very limited studies and documents can be found except for generic guidelines or review papers, and much less for DHOs’ COVID-19-related activities in dealing with the pandemic in the field.

## 2. Materials and Methods

A team consisting of those familiar with DHO work settings was created in April 2020. The team included researchers with public health backgrounds as well as physicians who manage organizational processes and are responsible for health care in the community.

The care pathway development started with a document review, including updates by the MOH, so as to better understand the public health measures practiced or conducted at the level of DHOs during the outbreak. Grey literature—including government reports, policy statements, and manuals of procedure—was searched. The *Malaysia Influenza Surveillance Protocol*, published in 2015 [14]; *Infectious Disease Outbreak: Rapid Response Manual* by the Disease Control Division, Ministry of Health Malaysia [37], and *Guidelines of COVID-19 Management in Malaysia* No. 04 and 05/2020, fourth and fifth editions [38,39], were the main points of reference. PubMed and ScienceDirect were among the electronic databases used in searching for published literature on COVID-19-related public health management.

The scope of this report is limited to public health measures, which are mostly non-pharmaceutical interventions used to reduce the spread of disease. We focus on how cases were managed at the district level from the perspective of the MOH as the public provider of health care. Within the organizational concept of a DHO, the workers perform their allocated tasks, covering a wide range of activities. The tasks cover a variety of disease prevention and control activities in outbreak management, extending from administrative tasks up to the point of contact for service delivery.

The type of public health measures implemented and the timing of service depend on the epidemiology of the virus and coordinated instruction within the MOH health care system. Therefore, the pattern of care delivered was reflected in the different phases. This report covers the services delivered from January until May 2020, in line with the country’s response to the COVID-19 pandemic. Within the designated timeframe, the activities were further split into three phases according to the phases of the Movement Control Order to control the pandemic, in which Phase 1 was the period before the MCO, from 16 January until 17 March 2020; Phase 2 was from 18 March until 14 April, constituting the first and second MCOs; while Phase 3 was from 15 April until 12 May—the third and fourth MCOs [24,25,26].

The latest shared information and instructions by the MOH, including the definitions of terms used, were taken into consideration [38]. The definition of a close contact in this report refers to either:

(i) Health-care-associated exposure without appropriate personal protective equipment, including providing direct care for COVID-19 patients, working with health care professionals infected with COVID-19, visiting patients, or staying in the same close environment as a COVID-19 patient; and/or
(ii) Working together in close proximity or sharing the same classroom environment with a COVID-19 patient; and/or
(iii) Travelling together in close proximity or sharing the same classroom environment with a COVID-19 patient; and/or
(iv) Living in the same household as a COVID-19 patient [38].

A confirmed case is defined as “a person with laboratory confirmation of infection with the COVID-19” [38] while a “person under investigation (PUI)” is a case with “Fever OR acute respiratory infection (sudden onset of respiratory infection with at least one of: shortness of breath, cough or sore throat), AND travel to or resided in affected countries in the 14 days before the onset of illness OR close contact in 14 days before illness onset with a confirmed case of COVID-19” [38] “OR Attended an event associated with known COVID-19 outbreak” [39]. The specific term of person under surveillance (PUS) was built up over time from these definitions, where the group is formed from the contacts of positive cases, travelers, and other targeted screening cases [39,40].

The flow of activities was described in the form of process map. The starting point was from attaining notification of a case, up until that case was released from surveillance. Throughout the outbreak investigation activity, the notified cases were managed according to the defined PUI and PUS terms. The activities were listed regardless of the source of the materials used to perform the tasks, which could come from either donation, personal purchasing, or central distribution. Then, these activities were linked to one another following the sequence of outbreak investigation steps. The activities were incorporated into algorithms of the COVID-19 outbreak response activity flow. These activities were classified so as to represent different kinds of intervention. There were tasks catering to different types of people with different needs. In contrast to hospital services, the prevention and control interventions cover various facilities and extend to the community level. Some essential services did not belong specifically to any of the activity flow groupings, but rather were present throughout the flow to complement the public health services as a whole, and appeared to stand alone. All activities were grouped into two main action areas: either service mainly for community care and case management, or activities requiring more administrative tasks, centered at the DHO level. The flow of activities was then further categorized according to the category of cases and level of care at which they were performed. Additional flow of activities branched out from the initial work process, and continued along its subsequent pathways in Phases 2 and 3. The activity flow algorithms were amended immediately following discussion. This method was chosen so as to fit the workers’ busy working schedules amidst the ongoing outbreak. The consensus between research team members and stakeholders further verified the pathway.

The discussion among the team members were initially focused on the first phase. Then, the pathways served as templates for the subsequent discussion, in which the stakeholders were engaged. The content was presented to representatives from various categories of personnel, including public health specialists, family medicine specialists, medical and health officers, assistant environmental health officers, nursing unit staff, assistant medical officers, and their respective supervisors and other assistant officers, counsellors, pharmacists, and clerks who worked in Klang DHO. The site was specifically selected for logistical reasons. The multi-professional team was briefed about the objectives and expectations in the initial stage. The researchers moderated the discussion.

## 3. Results

### 3.1. Care Pathway for Managing COVID-19

**Figure 1:**
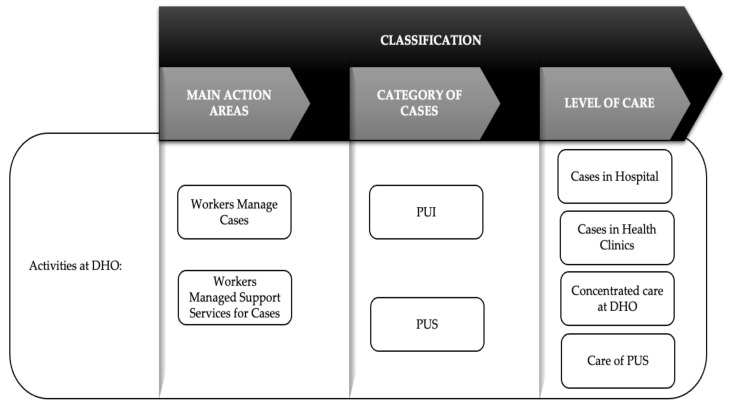
Diagrammatic view of classification of the activities.

The outbreak response activities can be broadly classified into three different sections: the main action areas, the category of cases, and the level of care. A diagrammatic view of the classification used in this report is illustrated in Figure 1. Case management and other support services that went hand in hand or were used in parallel during outbreak management at the DHO level formed the two main branches of action area. Additionally, the case management was further described according to the tasks of identifying PUIs for further consolidated clinical care, as well as the tasks of containing the spread of the disease—mainly from high-risk groups, namely, the PUSs. The tasks were managed at different levels of care, which were at the level of “PUI from hospital”, “PUI in health clinic”, “concentrated at DHO”, or “care for PUS”. Meanwhile, the support activities included human resource management, utilities and maintenance, transportation, outbreak operational room tasks, health promotion, sanitization activities, and deployment to the field to provide assistance, among others. Table 1 shows descriptions of activities across phases according to the classifications.

**Figure 2:**
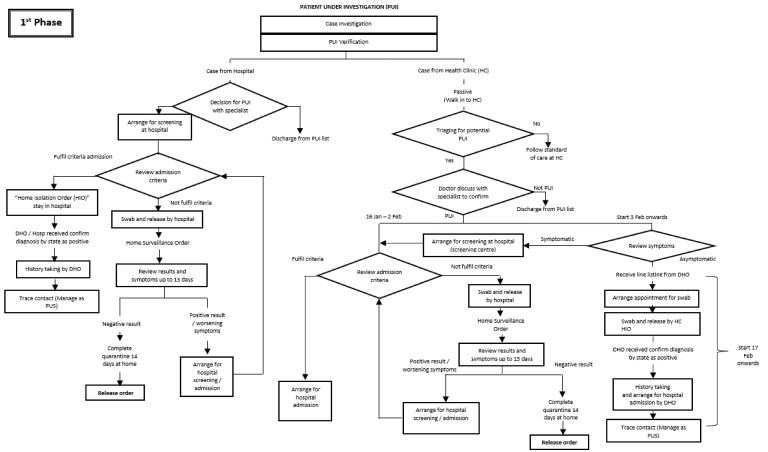
Managing PUIs during the first phase.

**Figure 3:**
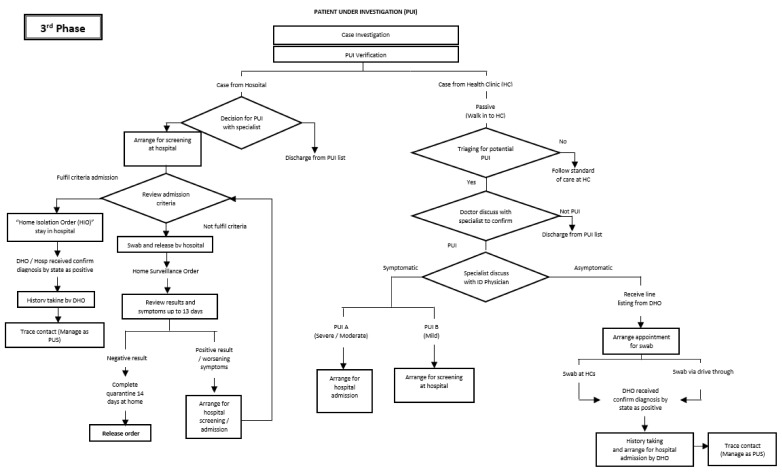
Managing PUIs during the third phase.

**Figure 4:**
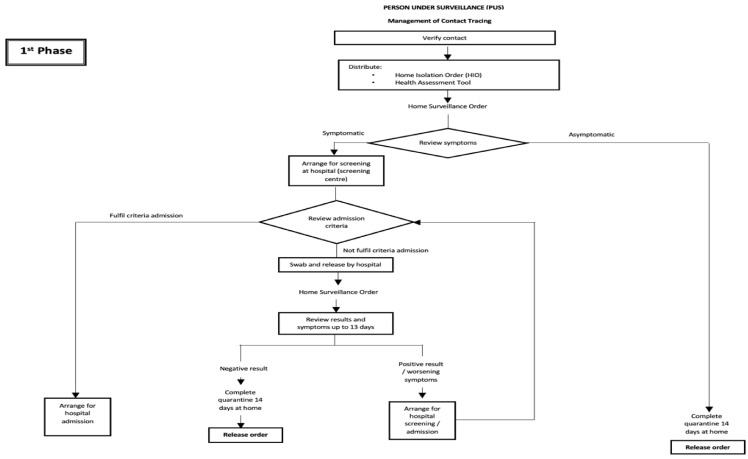
Managing PUSs (contacts) during the first phase.

The flow of activities is reflected in Figure 2, Figure 3 and Figure 4. Figure 2 illustrates the activities involved in managing PUIs during the first phase. The change in the flow of activities in managing PUIs during the third phase was as depicted in Figure 3. Figure 4 shows the flow of activities to manage contacts as PUSs in the early phase.

### 3.2. Progression of Care Pathway

As the outbreak progressed, the pattern of activities changed. The activities within the respective phases continued and expanded from Phase 1 to Phases 2 and 3, in keeping with the current state of the outbreak. Table 2 summarizes the changes noted in terms of the introduction or intensity of certain outbreak management activities.

## 4. Discussion

The occurrence of the COVID-19 outbreak highlights the importance of a strong outbreak management system in the country, and this is demonstrated at the national, state, and district levels of the MOH [14]. A majority of the action on the ground took place under the purview of a DHO, although public health responses in a state of outbreak require extensive and coordinated work processes by health and non-health agencies at multiple levels [12,25].

This report describes the organization of care processes with respect to the essential steps in control and prevention tasks during the COVID-19 outbreak in the community. Public health measures at the district level during the early stage of the pandemic were drawn from a predetermined function of DHOs and hospitals, where the prevention and control measures for community care were mainly performed by the DHO, while the more comprehensive focus of clinical care was mainly consolidated at assigned public hospitals. A definite line of command and coordination of resources reflects a well-structured government approach during a pandemic [25].

This paper distinctively describes the tasks of DHOs in response to the pandemic (Table 2). In the early stages of the outbreak, Malaysia escalated its national preparedness response through public health measures and compulsory hospital admission regardless of disease severity. As more clinical and public health information came to light, the standard operating procedures or guidelines were constantly updated and disseminated by the MOH, while health promotion activities became more focused. Multi-agency participation was intensified and resource management potentially became more challenging. Capacity building and counselling support similar to those in a hospital setting [40] were encouraged for the wellbeing of the DHO workers [3,18].

More types of high-risk groups and PUS categories were identified by the DHO as the phases progressed. This was in accordance with the specific guidelines of the MOH acknowledging special settings with higher risk for transmitting COVID-19 [39]. These groups include interstate travelers, the homeless, occupants of care homes, preoperative patients or those awaiting any other procedures, and those awaiting entrance to universities, colleges, or madrasah. These guidelines resulted in a more targeted intervention, whereby sampling activities were initially performed by assigned health care workers coordinated by DHOs, but were later expanded to health clinics.

According to the World Health Organization, contact tracing that is performed correctly and applied systematically should result in breaking the chains of transmission of any infectious disease and reducing transmission within clusters. Hence, contact tracing is an essential tool in the control of the COVID-19 pandemic [41]. Contact tracing was crucial in the case management of both PUIs and PUSs. Most secondary infections were among household contacts, where several studies have documented a secondary attack rate of up to 15% among this group of contacts [42,43,44]. Quarantine centers were established during the second phase. The WHO has decreed that any countries that choose to implement mandatory quarantine for all travelers on arrival should do so based on their own’ risk assessment and local circumstances [45].

DHOs adapted the national operational plan for local use, guiding health care workers in conducting tasks and in monitoring any health situations. Direct daily fact-based communication between workers within and across other DHOs enhances understanding and co-operation. Tasks were performed collectively at respective DHO levels. Accordingly, daily press conferences on risk communication in the management of COVID-19 in Malaysia at the ministerial level are fed by the crucial inputs from districts [26,46].

In this report, it was found that the progression of the pandemic translated directly to changes in the pattern of activities, with additional subgroups of activities so as to comply with newly imposed guidelines and policies. These activities were seen to be linked to one another and distributed within the DHOs’ operational boundaries. Simultaneously, the administrative and other support activities were maintained throughout, and were seen to complement the case management activities. On another note, the description of activities was uniform regardless of the health care workers’ positions or their job categories.

The pathway described in this report demonstrates the commitment from managers and good communication by the “hands-on” workers from multidisciplinary units within the public health discipline. Clear and systematic management care is pivotal in managing any kind of outbreak. However, this care pathway is not fixed, but evolves as the pandemic progresses, allowing flexibility in the outbreak management.

The phases selected in this report were appropriate, as they were in line with the national strategy to battle an unpredictable pandemic in the community at that time. Indisputably, the ongoing pandemic poses a significant challenge to the team members. The verification process with experts at the site was prolonged due to the difficulty of finding the time for consultation, because of their busy schedules managing the continuing pandemic. Only some parts of the pathways are enclosed as figures, as an illustration to help better understand the work done at the DHO level.

Activities outside the scope of a DHO administrative role, such as laboratory testing for COVID-19—the management of which was confined to the National Public Health Laboratory—or collaboration between other public health authorities or civil society that did not actively involve the DHOs, were excluded. The direct impact of MCO strategies on the tasks at DHOs or the quality of the work system—such as auditing for compliance to guidelines—was also not assessed. As part of a holistic government’s efforts in controlling the pandemic, it is important to acknowledge that the pattern of care practiced by other DHOs elsewhere may be influenced by the geographical distribution of facilities assigned for COVID-19-related care, or by adaptations made in the management of a DHO.

Apart from that, the magnitude of resources consumed for the activities may vary according to the duration of time for the event to occur, management style, number of workers, cases, or patient load, as well as the country’s imposed local or national strategy. As more resources are expected to be utilized and managed during the pandemic, more complex activities were assumed in different timeframes. Thereafter, the value of the resources can be estimated using a standardized format. Nevertheless, quantifying the identified resources or evaluating the pathway implementation is beyond the scope of this report.

## 5. Conclusions

In combating the COVID-19 pandemic, public health measures operationalized at DHOs varied according to local occurrences and the new policies, standard operating procedures, and instructions from the MOH.

Algorithms in the care pathway map the complexity of services delivered throughout the duration of the pandemic. Moreover, activities in the tasks performed by the workers within this five-month period of the COVID-19 pandemic also evolved. The relative differences in the activities between the first and third phases were notable. The pathways help to visualize the work process, and may serve as templates in another study at the district level, or be used in other quality improvement initiatives. With the improvements in the flow of activities and processes throughout the phases, the COVID-19 situation in the country was subsequently improved.

In their current form, the pathways are non-exhaustive, and could be complemented in a separate study by adding further information, such as human resources through retrospective review of records. Generally, the pathways were described so as to fit the flow of the activities into a set of study frameworks, in fulfilment of the central role of a DHO. This will be followed in the future by estimation of the resources of public health measures for COVID-19, and subsequently used to calculate the costs from the perspective of DHOs.

## Figures and Tables

**Table 1:** Care pathways in public health activities.

Classification		Activities in Different Phases		
		Phase 1	Phase 2	Phase 3
A. Administrative and other support activities	i. Concentrated at DHO	Human resource management started with a systematic operation room organization chart headed by a commander; Operation room was set up to coordinate all intra- and inter-agency activities, handling of risk communication, preparing the daily report, and to prepare information to be disseminated to relevant parties or stakeholders; Meetings, briefings, or discussions and data management activities specifically to prepare line listings and update datasheets; Timetables and rosters were drawn to organize the staff; Management of personal protective equipment (PPE) and other consumables; Health promotion activities to disseminate information or educate the population; Risk management activities including report writing, information dissemination, and answering queries from the public; Training and counselling for staff.	Similar activities as in Phase 1	Similar activities as in Phase 1
B. Case Management	i. PUIs from hospitals	For PUIs from hospitals, they were either admitted, or released home after the sampling procedure and served the HIO (HIO: home isolation order). History was taken by a DHO officer and patients’ contacts were traced; Those discharged home were monitored for 14 days and their COVID-19 results were traced; Those symptomatic or whose results came back as positive for COVID-19 were admitted, while those who remained asymptomatic with negative results were served a release order to release them from quarantine at day 14 (Figure 2).	Similar activities as in Phase 1	Similar activities as in Phase 1
	ii. PUIs in Health Clinics	Initially, PUIs who fulfilled the admission criteria were sent straight to the assigned public hospital, where they were either admitted or released home after completing the sampling procedure. Starting from 3 February 2020, symptomatic PUIs from clinics were sent to a screening public hospital, where staff would review the hospital admission criteria and decide on whether to admit or release for home quarantine after a COVID-19 sample was taken; For those who were released home, DHO officers monitored their symptoms for 14 days, traced their results, and arranged for admission or released them from quarantine accordingly. Starting from 17 February 2020, sampling procedures for the asymptomatic PUIs were carried out at health clinics; A line listing was provided by the DHO, and cases were given appointments to come for the sampling procedure. They were subsequently served with HIOs and released home; History taking and contact tracing was carried out by the DHO health officers for all positive cases once informed of the result by the State Health Department.	Upon the diagnosis of PUIs in health clinics, specialists from health clinics and hospitals would categorize symptomatic PUIs as PUI A for severe-to-moderate cases and PUI B for mild cases; For PUI A, DHO would arrange for admission, while those who fall under PUI B would be sent for screening at assigned hospitals; The flow for asymptomatic PUIs was alike for all phases.	Similar activities for symptomatic PUIs in Phase 2; Additional facility for “drive-through” sampling for asymptomatic PUIs under one of the health clinics (Figure 3).
	iii. PUSs - Contacts of positive cases	Contacts were verified, served the HIO, and symptoms monitored for 14 days. The asymptomatic were served the release order to discharge them at day 14; Those who were symptomatic were sent to a screening hospital, where their criteria for admission were reviewed; Those who fulfilled the hospital admission criteria were admitted, while those who did not were swabbed and released home to complete their 14 days of home isolation; The DHO continued to monitor their symptoms and traced the COVID-19 results. Those with positive results were admitted; The cycle continued until they were deemed fit to be served the release order upon completion of their home isolation order (Figure 4).	Similar activities for asymptomatic contacts as in Phase 1. For symptomatic contacts, they were categorized into two groups: the mild or moderate group, and the severe group; Those in the mild or moderate group were given appointments for sampling procedure at designated health clinics, where their samples were sent to the Public Health Laboratory in Sungai Buloh or to the Institute for Medical Research; Cases in the severe group were sent to the screening hospital after discussion with the infectious disease physician; Those who fulfilled the admission criteria were admitted, while those who did not were discharged after serving them the HIO. The DHO monitored their symptoms and traced their results for 14 days; Contacts with negative COVID-19 results were booked in for a second sample at day 13; Cases with positive results were admitted, while a release order was issued if they remained negative throughout the 14-day home isolation order.	Contacts were given appointments for sampling upon identification, in addition to serving them with the HIO and the home assessment tool for symptom monitoring; Those who were asymptomatic and whose swab results came back as negative for COVID-19 were allowed to complete home isolation for 14 days, while contacts who were symptomatic or whose swab results came back as positive were discussed with the infectious disease physician with regard to the admission criteria; Contacts who fulfilled the admission criteria were admitted, while those who did not were either sent to a quarantine center or discharged to complete the home isolation order at their own home; The DHO monitored their symptoms and traced the results for 14 days. Those with positive COVID-19 swab results or worsening symptoms were admitted, while those with negative results were subjected to a second sampling procedure using a rapid test kit (RTK) at day 13. Positive RTK results had to be confirmed by swabs. Those with positive swab results were admitted, while those with negative results by either RTK or swab would continue their home isolation order for 14 days before being served with the release order.
	- Travelers	Travelers to Malaysia entering the country through KLIA (KLIA: Kuala Lumpur International Airport) were screened for symptoms; Those symptomatic within 14 days were swabbed and sent for admission, while those who remained asymptomatic throughout the 14 days were discharged as PUSs. The DHO deployed its officers to KLIA to provide assistance.	Similar activities as in Phase 1; However, those asymptomatic were required to be quarantined in a designated quarantine center, initially all within KLIA. The first quarantine center under Klang DHO commenced on 5 April 2020. Similarly, in quarantine centers, symptoms were monitored for 14 days and results were traced; Symptomatic or positive COVID-19 cases were admitted, while those with negative results and who remained asymptomatic throughout were released after the completion of day 14.	RTKs were used for the second sampling procedure at day 13 in quarantine centers. Positive RTKs warranted a repeat swab to be taken, and only those with positive results from the swabs would be sent for admission; Those who remained negative for all procedures were served the release order at day 14; Management for travelers and quarantine center groups were similar, because travelers were the majority of occupants in the quarantine centers, in addition to several other smaller groups of people who were unable to complete quarantine at their own homes.
	- Targeted Screening	NA (NA: not applicable)	NA	Other high-risk groups were identified, including the homeless; the elderly in care homes, Tahfiz centers, or institutions; and food delivery service workers. A line list for the groups was obtained prior to the sampling activity. An outreach program was held at care homes and among the homeless for symptomatic screening and sampling procedures; Those who were symptomatic, or whose results came back as positive for COVID-19, were arranged for admission, while those who were asymptomatic with negative results were discharged from the PUS list; As for the students in schools, and food handlers, a similar line list was prepared at the DHO. The groups were then contacted and given appointments for sampling at a health clinic; Results were traced, and subsequent admission was arranged for those with positive results, while those with negative results were discharged from the PUS list.

**Table 2:** Evolution of the main activities over different phases.

Activities	Phases		
	Phase 1	Phase 2	Phase 3
Managing outbreak	Outbreak preparedness team activated.	Case investigation, contact tracing, enforcement, and surveillance activities strengthened.	Same as Phase 2.
Sampling activities	Screening activities consolidated at the hospital level.	Health clinic involvement in screening activities.	Extension of screening to more health clinics; Drive-through sampling for screening.
Data management	Manual data entry.	Same as Phase 1.	Usage of *e-COVID* electronic information system.
High-risk group	Focus of control activity on one type of high-risk group.	Same as Phase 1.	Expansion of high-risk group categories.
Quarantine	DHO responsible for cases quarantined at home.	Quarantine centers under the DHO established.	Similar activities in quarantine centers as Phase 2.
Mobilization of staff	Mobilized mainly to KLIA and some to HSB (HSB: Hospital Sungai Buloh).	Mobilized mainly to HSB and some to KLIA.	Mobilized mainly to other DHOs; only some to KLIA.
Health promotions	Usual activities.	Health promotion activities inclusive of topics relating to COVID-19.	Focused activities on COVID-19.
Multi-agency participation	Usual collaboration activities.	Multi-agency collaborations enhanced.	Same as Phase 2.
Counselling for workers	Usual counselling activities.	Health care worker counselling support related to COVID-19 promoted and encouraged.	Dedicated counselling focused on COVID-19 cases.
